# The effects of feeding diets with ensiled maize grain on the production efficiency and meat characteristics of broiler ducks

**DOI:** 10.2478/jvetres-2026-0020

**Published:** 2026-04-16

**Authors:** Bartosz Bigorowski, Sebastian Wlaźlak, Izabela Narloch, Szymon Różański

**Affiliations:** 1 Department of Animal Breeding and Nutrition, Laboratory of Chemical Research and Instrumental Analysis, Bydgoszcz University of Science and Technology, 85-084, Bydgoszcz, Poland; 2 Department of Food Analysis and Environmental Protection, Bydgoszcz University of Science and Technology, 85-326, Bydgoszcz, Poland; 3 Laboratory of Chemical Research and Instrumental Analysis, Bydgoszcz University of Science and Technology, 85-084, Bydgoszcz, Poland

**Keywords:** ducks, performance, digesta viscosity, meat quality, fatty acid profile

## Abstract

**Introduction:**

Alternative methods of duck feeding can be good solutions for reducing production costs on small farms. Ensiled corn grain is a good source of energy that can be used in the rearing of broiler ducks. This research investigated productivity effects and meat quality parameters in experimental broiler ducks provided this material as feed portions.

**Material and Methods:**

Broiler ducks were divided into a control group (CON) and two experimental groups (CSMIX and CD+SG). In the CSMIX group, birds were fed a commercial diet mixed with ensiled corn grain in a ratio of 80:20. In the CD+SG group, the commercial diet was limited, and ensiled corn grain was *ad libitum*.

**Results:**

The highest body weight and weight gain were found in the CSMIX group. A lower feed intake was found in the control and CD+SG group than in the CSMIX group throughout the rearing. A higher final feed conversion ratio characterised ducks from the experimental groups and a higher pectoral muscle and total muscle weight than the CD+SG group was noted in carcasses of ducks from the CON group. In the pectoral and leg muscles of the CON and CSMIX groups, the content of polyunsaturated fatty acids was higher than in these muscles of the CD+SG group.

**Conclusion:**

Duck feeding with ensiled corn grain (80:20) could be a feeding strategy for small farms because it yields favourable growth parameters, carcass composition and high polyunsaturated fatty acid content in meat, and reduces feed costs.

## Introduction

Alternative duck feeds are mainly given on small farms, where plant raw materials are readily available. This allows breeders to reduce production costs and control the quality of raw feed materials (6). Developing an innovative feeding strategy for waterfowl production could have tangible benefits, as feed costs can account for up to 70% of the total production costs (60).

The specificity of the duck digestive tract promotes the digestion of feeds with a higher dietary fibre content. This allows the possibility of feeding roughage to this poultry species (52). Therefore, in the study by Zaremba *et al*. (64), nutrition cutting out some commercial diet and substituting silage from beet pulp and whole maize plants *ad libitum* was provided. The authors noted that the experimental strategies yielded more profit per bird than the control groups. Kierończyk *et al*. (24), found changes in the pH of the gizzard, ileum and cecum contents of Pekin ducks fed with chemically preserved and high-moisture whole maize at levels of 5 and 10%. Wang *et al*. (57), showed that whole-maize plant silage in goose rearing affected the microbial composition and abundance of certain types of bacteria. Kokoszyński *et al*. (25), tested the effect of wholemaize silage provided *ad libitum* to White Kołuda geese from the 22^nd^ to the 98^th^ day of life. In the experimental group (silage), a significantly higher slaughter yield and proportion of pectoral and leg muscle mass to whole carcass mass were found in 17-week-old geese. The results confirmed that alternative feeding strategies affected production economics, carcass composition, and birds’ gastrointestinal tract microflora.

After harvesting, maize grain is characterised by a high water content – up to 50%. It is necessary to dry or preserve it to extend the storage time. Dried maize grain is an essential energy raw material in poultry feed production (31). However, ensiling is a cheaper preservation method, which maintains a high nutritional value (7). This grain is characterised by a lower content of nitrogen-free extract compounds, a higher protein content and higher digestibility of nutrients (15). Although maize grain silage is common in cattle and pig nutrition and has been well studied (21, 62), information on its effects in waterfowl nutrition is limited. The study aimed to explore the possibility of using ensiled maize grain to improve production results, carcass characteristics, meat quality and the profitability of Cherry Valley broiler duck production.

## Material and Methods

The study was carried out adhering to current legislation on the protection of animals used for scientific or educational purposes (13, 17). The Committee for the Care of Animals (Local Ethics Committee) at Bydgoszcz University of Science and Technology in Poland approved the test procedures and methods (Approval No. 1/24), which were also compliant with the animal research: reporting of *in vivo* experiments (ARRIVE) guidelines (33).

### Animals and experimental design

In the experiment, 180 one-day-old male Cherry Valley broiler ducks were kept for 49 days. The birds were purchased from a commercial duck hatchery (DROB, Tulce, Poland). The birds were divided into three groups of 60 and each group was divided into six replicates. The size of each pen was 1.44 m^2^, and each was made of stainless steel frames and mesh. Wheat straw was used as bedding. On the first day, the temperature in the building measured at the height of the ducklings was 28°C. Then, it was lowered gradually to fall to 20°C in the 4^th^ week of rearing. During the first four weeks of rearing, the ducks had access to an additional heat source (30–32°C). Other microclimate parameters were consistent with those established by Biesek *et al*. (6). The ducks had access to water and *ad libitum* feed throughout the experiment. The birds were provided with nipple drinkers (two per 10 ducks) and feeders (with a length of 7 cm per duck). Feeding was divided into two periods: starter feed was provided from day 1 to day 28 and grower feed from day 29 to day 49. Three feeding groups were reared: the CON group, which received 100% granulated commercial diet *ad libitum*; the CSMIX group, which received a mixture of 80% granulated commercial diet and 20% ensiled corn grain, administered *ad libitum*; and the CD+SG group, which received a limited amount of granulated commercial diet along with ensiled corn grain *ad libitum*. The ensiled corn had a smaller grain size than the commercial diet. The CD+SG group were left a free choice between the two offered feeds. For this purpose, double feeders were used, one of which contained commercial diet, and the other of which contained ensiled corn grain. The proportion reduction in the daily amount of commercial diet was established based on data previously reported by Zaremba *et al*. (64). In the CD+SG group, only the commercial feed was given for the first three days. Subsequently the following amounts of commercial feed were given per pen (10 birds): 251.75 g (days 4–7), 528.00 g (days 8–14), 1,020.00 g (days 15–21), 1,735.71 g (days 22–28), 2,201.86 g (days 29–35), 2,337.14 g (days 36–42) and 2447.71 g (days 43–49).

### Feed composition

Based on the manufacturer’s declaration, the starter feed contained maize, wheat, soybean extraction meal, wheat bran, sunflower extraction meal, hulled sunflower seeds, barley, rapeseed extraction meal, wheat gluten feed, calcium carbonate, animal fat, monocalcium phosphate, vegetable oil and fat (raw sunflower), sodium chloride, and sodium sulphate. The crude protein proportion was 19.30%, that of crude fat was 3.90%, crude fibre was 4.40%, crude ash 5.20%, lysine 0.94%, methionine 0.44%, calcium 0.60%, phosphorus 0.61% and sodium 0.16%. The grower feed contained maize, wheat, wheat bran, soybean extraction meal, sunflower extraction meal, from dehulled sunflower seeds, triticale, rapeseed extraction meal, animal fat, calcium carbonate, monocalcium phosphate, sodium chloride and calcium bicarbonate. Crude protein made up 17.20%, crude fat 3.90%, crude fibre 4.90%, crude ash 4.70%, lysine 0.85%, methionine 0.36%, calcium 0.60%, phosphorus 0.56% and sodium 0.15%.

### Ensiled corn grain production

Corn grain of the Pioneer 8500 variety with a moisture content of 34% was ensiled. Twenty-four hours after harvesting, the grain was ground in Gruber machinery (Gaspoltshofen, Austria). The Agro-Sil Corn ensiling additive (Agrifood, Bojanowo, Poland) was added in a 3 kg mass per 1,000 kg of grain. The additive contained propionic acid, formic acid, lactic acid, ammonium formate, propionate, anti-corrosive substances and a carrier. The prepared mixture was next tipped into plastic bags with a capacity of 1,000 kg and compacted by rapid lifting and lowering, and the bags were tightly sealed after 24 h to limit access to oxygen. Ensiled corn grain was given to the ducklings 32 weeks after the bag was closed.

### Chemical composition of diets

A POL-EKO dryer (Wodzisław Śląski, Poland) was used to calculate the dry matter content with the technique stipulated in the Polish Animal feeding stuffs - determination of moisture and other volatile substances standard PN-ISO 6496:2002 (35). The analysis was carried out using the weight method, similarly to the analysis of crude ash content (10). The crude fat content was assessed using a Soxtec System HT 1043 extraction apparatus (FOSS Tecator, Hilleroed, Denmark), in observance of the Polish Animal feeding stuffs - determination of fat content standard PN-ISO 6492:2005 (37). The Kjeldahl method determined the crude protein content using the Kjeltec 8400 analyser and the Kjeltec 8420 sampler (FOSS). This analysis followed the method in the PN-EN ISO 20483:2014-02 standard, considering a factor of 6.25 (43). The content of acidic detergent fibre (ADF) was assessed by weight, including residual ash and acid-detergent lignin (ADL) (40) and neutral detergent fibre (NDF) (39) using an ANKOM 220 fibre analyser (Ankom, Macedon, NY, USA). Gross energy was determined, using a KL-21 PLUS isoparabolic calorimeter (Precyzja-Bit, Bydgoszcz, Poland) and adhering to the Polish Animal feeding stuffs, animal products, and faeces and urine - determination of gross energy – bomb calorimetric method standard PN-EN ISO 9831:2005 (38). The above analyses were carried out for commercial diets, commercial diet mixtures with ensiled corn grain (80:20 ratio) and ensiled corn grain, and for both rearing periods. Samples of starter feed were taken on day 1 and samples of grower feed were collected on day 29 (grower feed) – three samples of each type of feed/silage in two replicates. Gross energy was calculated in MJ/kg of dry matter and other nutrient values in kg of dry matter. The chemical composition of feed and ensiled corn grain is shown in Table 1.

**Table 1.** Chemical composition of broiler duck feed and ensiled corn grain

Ingredient^1^	Starter diet	Grower diet	Corn grain silage	80% starter diet and 20% silage	80% grower diet and 20% silage
Dry matter (g/kg feed)	895.03	892.00	601.70	836.37	833.94
Crude ash (g/kg dry matter)	53.91	50.13	26.48	48.42	45.40
Total protein	219.71	200.51	142.61	204.29	188.93
Crude fat	37.24	36.71	64.82	42.75	42.33
Crude fibre	49.89	53.66	104.54	60.82	63.59
ADF	294.22	77.87	383.29	312.04	138.96
NDF	390.88	274.27	548.09	422.32	329.04
ADL	97.94	15.73	101.44	98.64	32.87
Gross energy (MJ/kg dry matter)	18.64	18.63	28.83	20.68	20.67

1 Mean values are presented based on 6 replicates for each type of feedstuff;

1 – ADF, acid detergent fibre; NDF – neutral detergent fibre; ADL – acid detergent lignin

### Fatty acid profile and vitamins of feed and muscles

For the determination of vitamins E (α-tocopherol and β+γ tocopherol) and A (retinol), samples were prepared according to Polish standards (36, 42) with modifications. The duck meat and feed samples were homogenised and saponified. The next step was extraction using a mixture of n-hexane and ethyl acetate. After separating layers, the supernatant was collected into chromatographic vials and evaporated to dryness under nitrogen. The residue was reconstituted with 1 mL of solvent and filtered through a 0.45-μm PTFE filter disc into two chromatographic vials for simultaneous analysis by liquid chromatography with UV and fluorescence detector (UFLC HPLC System; Shimadzu, Canby, OR, USA). Peak areas were manually integrated with LCsolution v. 1.25 (Shimadzu). Vitamin content was estimated using a standard curve obtained from various concentrations of standard solutions. The total vitamin content in the samples was calculated as the amount of vitamins in mass units of the sample.

Lipid extraction from feed and meat was carried out according to Folch *et al*. (14), with adaptations. Lipids were extracted from samples using chloroform and methanol (2:1, v/v). The mixture was homogenised, ultrasonicated and centrifuged. The chloroform layer was removed under a nitrogen stream. The obtained residue was transesterified and centrifuged, and the extract was collected. Finally, hexane with the fatty acid methyl esters was subjected to chromatography analysis using a 7890B gas chromatography system (Agilent Technologies, Santa Clara, CA, USA) equipped with a flame ionisation detector, autosampler and split/splitless injector. The retention times of fatty acid methyl ester (FAME) compounds for meat and feed samples were identified by comparison with a standard (FAME Mix-37; Sigma-Aldrich, St. Louis, MO, USA). Peak areas were manual integrated with Agilent ChemStation F.01.00.1903 (Agilent Technologies). The results were presented as a percentage of the total area of the chromatograms using the correction factors of the flame ionisation detector (5). The fatty acid profile and vitamin content in diets are presented in Table 2.

**Table 2.** Fatty acids profile and vitamins in broiler duck feed

Item	Starter diet (%)	Grower diet (%)	Corn grain silage (%)	80% starter diet and 20% silage (%)	80% grower diet and 20% silage (%)
C10:0	0.038	0.035	0.097	0.037	0.047
C12:0	0.298	0.358	0.213	0.310	0.329
C14:0	0.703	0.670	0.875	0.696	0.711
C14:1n-5	0.113	0.108	0.110	0.112	0.108
C15:0	0.122	0.118	0.168	0.121	0.128
C16:0	14.097	14.450	12.083	14.168	13.977
C16:1n-7	0.938	1.292	0.357	1.009	1.105
C17:0	0.140	0.143	0.193	0.141	0.153
C17:1n-7	0.088	0.105	0.077	0.091	0.099
C18:0	3.263	3.052	2.778	3.221	2.997
C18:1n-9t	25.138	26.538	24.783	25.418	26.187
C18:1n-9c	1.268	1.760	0.720	1.366	1.552
C18:2n-6t	0.000	0.000	3.412	0.000	0.682
C18:2n-6c	48.787	46.010	49.533	48.232	46.715
C18:3n-3	2.877	3.097	0.715	2.921	2.621
C20:0	0.307	0.285	0.505	0.303	0.329
C20:1n-9	0.575	0.638	1.837	0.588	0.878
C20:2	0.122	0.158	0.053	0.129	0.137
C20:3n-3	0.035	0.048	0.000	0.038	0.038
C20:3n-6	0.073	0.082	0.000	0.075	0.066
C20:4n-6	0.125	0.153	0.085	0.131	0.139
C20:5n-3	0.000	0.028	0.000	0.006	0.022
C21:0	0.047	0.055	0.218	0.049	0.088
C22:0	0.288	0.223	0.182	0.275	0.215
C22:2	0.078	0.102	0.450	0.083	0.172
C22:6n-3	0.125	0.127	0.123	0.125	0.126
C23:0	0.062	0.055	0.032	0.061	0.050
C24:0	0.195	0.173	0.215	0.191	0.181
C24:1n-9	0.107	0.127	0.152	0.111	0.132
SFA	19.557	19.623	17.558	19.570	19.210
MUFA	28.285	30.567	28.067	28.741	30.067
PUFA	52.095	49.933	54.352	51.663	50.817
TFA	25.138	26.538	28.193	25.418	26.869
α-tocopherol μg/100g	2752.495	2264.428	161.398	2654.882	1843.822
β+γ tocopherol μg/100g	1567.155	1126.695	1069.895	1479.063	1115.335
β-carotene μg/100g	31.695	17.580	117.667	28.872	37.597
Retinol μg/100g	173.805	130.527	14.370	165.149	107.296

1 C16:0 – hexadecanoic acid; C16:1n-7 – cis-9-hexadecenoic acid; C17:0 – heptadecanoic acid; C17:1n-7 – cis-10-heptadecenoic acid; C18:0 – octadecanoic acid; C18:1n-9t – trans-9-octadecenoic acid; C18:1n-9c – cis-9-octadecanoic acid; C18:2n-6t – trans-9,12-octadecadienoate acid; C18:2n-6c – cis,cis-9,12-octadecadienoic acid; C18:3n-3 – cis,cis,cis-9,12,15-octadecatrienoic acid; C20:0 – arachidic acid; C20:1n-9 – cis-11-eicosenoic acid; C20:2 – cis-11,14-eicosadienoic acid; C20:3n-3 – cis-11,14,17-eicosatrienoic acid; C20:4n-6 – arachidonic acid; C20:5n-3 – eicosapentaenoic acid; C21:0 – heneicosanoic acid; C22:0 – behenic acid; C22:2 – docosadienoic acid; C22:6n-3 – cis-4,7,10,13,16,19-docosahexaenoic acid; C23:0 – tricosanoic acid; C24:0 – lignoceric acid; C24:1n-9c – nervonic acid; SFA – saturated fatty acids (C10:0, C12:0, C14:0, C15:0, C16:0, C17:0, C18:0, C20:0, C21:0, C22:0, C23:0 and C24:0); MUFA – monounsaturated fatty acids (C14:1n-5, C16:1n-7, C17:1n-7, C18:1n-9t, C18:1n-9, C20:1n-9 and C24:1n-9); PUFA – polyunsaturated fatty acids (C18:2n-6t, C18:2n-6c, C18:3n-3, C20:2, C20:3n-3, C20:4n-6, C20:5n-3, C22:2, C22:6n-3 and C24:6n-3); TFA – trans fatty acids (C18:1n-9t and C18:2n-6t)

### Growth performance

Birds were weighed on days 1, 28 and 49 (Radwag, Radom, Poland) with an accuracy of ±0.01 g. The amount of feed and leftovers was controlled. Feed intake (FI), body weight gain (BWG), growth rate (GR), feed conversion ratio (FCR) and European broiler index (EBI) were calculated. Bird deaths were recorded in each group, and viability (%) was calculated. The formulas for the parameters mentioned above are as follows:

$$\begin{array}{l} \text{BWG }=\text{ final body weight }(\text{g})-\text{initial body weight }(\text{g})\\ \text{GR}=\frac{\text{ final body weight }(\text{g})-\text{initial body weight }(\text{g})}{0.5\times (\text{ initial body weight }(\text{g})+\text{final body weight }(\text{g}))}\times 100\%\\ \text{FCR}=\frac{\text{FI}(\;\text{kg})}{\text{BWG}(\;\text{kg})}\\ \text{EBI}=\frac{\text{viability }(\%)\times \text{average daily gain}\left(\frac{\overset{g}{\bar{chick}}}{day}\right)}{\mathrm{FCR}\left(\frac{\text{kg feed }}{\text{kg gain }}\right)\times 10} \end{array}$$

### Carcass composition

On the last day of rearing, 12 selected males from each group were weighed, and 2 from each pen with body weight close to the average of their penmates were selected. They were sacrificed by stunning with an electrical device following the recommendations (13) and then decapitation. The birds were plucked with a mechanical plucker (Soda Plus, Krępice, Poland). Then, the carcasses were placed in melted wax approved for contact with food for 3 s (Polwax, Jasło, Poland) and cooled in cold water. Wax and feather remains were removed, and the carcasses were eviscerated. The heart, liver and gizzard were collected. Carcasses and offal were cooled at 4°C in a refrigerator (Hendi, Poznań, Poland) for 24 h. Before dissections were performed, the carcasses and offal were weighed. The following were dissected and separated: neck without skin, pectoral muscles (*m. pectoralis* major and minor), leg muscles (drumsticks and thighs without bones), skin with subcutaneous fat (including neck skin), abdominal fat, wings with skin and carcass remains (trunk and leg bones) (65).

### Digesta viscosity

The digesta viscosity was analysed based on a modified method presented in the study by Pestana *et al*. (34). After the carcasses had been eviscerated, six samples of jejunal and ileal digesta (approximately 7.5 mL each) were taken from each group into 15 mL Falcon tubes. The samples were separated in a laboratory centrifuge (Eppendorf, Hamburg, Germany) at 4,200 rpm for 10 min. The supernatant (1.5 mL) was collected with an automatic pipette (Chemland, Kraków, Poland) and placed in Eppendorf tubes of the same capacity. A Brookfield DVNext viscometer (Middleboro, MA, USA) was used to analyse the digesta viscosity. The supernatant (0.5 mL) was poured onto a plate, and viscosity was measured at a speed of 6 rpm. The final result was the average of all the results obtained during 30 s of measurement. Each measurement was repeated giving 12 results per group. Results were recorded in centipoise (cP).

### Meat quality

The pH of the pectoral muscle (major) was measured using a pH meter equipped with a dagger electrode (Elmetron, Zabrze, Poland). The device was calibrated before it gave measurements using standard buffers with pH 4.00, 7.00 and 9.00. The colour of the pectoral and leg muscles was measured from their inner side. A colorimeter was used (Konica Minolta, Tokyo, Japan) with a three-point colour scale: L*–lightness, a* – redness, and b*–yellowness (11). The right pectoral muscle was weighed (M1) and placed in a cut string bag, which was itself placed inside a larger string bag. After 24 h at a temperature of 4°C, the samples were weighed again (M2), and the drip loss was determined. The water holding capacity of the pectoral and leg muscles was assessed after they were comminuted with a meat grinder (Hendi, Poznań, Poland). Samples weighing 0.300 ± 0.005 g (in M1) were measured. The muscles were placed between two sheets of Whatman tissue paper (4 × 4 cm) and compressed with a 2 kg weight for 5 min. The weight was then removed, the samples were removed, and the samples were weighed again (making M2), according to Grau and Hamm (18). Chemical composition analysis was performed in the pectoral and leg muscles to determine the content of protein, collagen, salt, intramuscular fat (IMF) and water. This was performed on three samples weighing approximately 500 g from each group in three replicates). Near-infrared transmission spectrophotometry was used with a FoodScan instrument (FOSS), working to the Polish Standard for Meat and meat products - determination of fat, protein, and water content - Near Infrared Transmission Spectrometry (NIT) using Artificial Neural Network calibration PN-A-82109:2010 standard (41).

### Statistical calculation

The mean values, SEM, homogeneity of the sample and normal distribution were analysed. A unidirectional ANOVA was conducted to evaluate the data, and Tukey’s test was used to identify statistically significant differences between groups at a significance level of P-value < 0.05. The data were analysed using Statistica software v. 13.3 (TIBCO, Palo Alto, CA, USA). The statistical model was *Ya* = μ + *Da* + *ea*, where *Ya* was the dependent variable, μ was the overall mean, *Da* was the effect of nutrition (a = CON, CSMIX or CD+SG) and *ea* was residual error.

## Results

### Growth performance

Significantly lower body weight on the 28^th^ day was noted in the CD+SG group compared to the CON and CSMIX groups (P-value < 0.001). On the 49^th^ day of rearing, significantly higher body weight was measured in the CSMIX group than in the CD+SG group, while the CON group did not differ significantly from either group. The lowest GR and BWG (in days 1–28) were found in the CD+SG group and this deficit was significant (P-value < 0.001). In the second feeding period, the values of GR and BWG were significantly higher in the CD+SG group than in the other groups (P-value < 0.001). Birds in the CSMIX group gained significantly more weight for the entire rearing period than birds in the CD+SG group (P-value = 0.047). The CON and CD+SG groups had significantly lower feed intake throughout the rearing period than the CSMIX group (P-value = 0.023). There was a significantly higher FCR throughout the entire rearing period in both experimental groups than in the control group (P-value = 0.048). A significantly higher EBI value was returned for ducks in the CSMIX group compared to those in the CD+SG group (P-value = 0.004) (Table 3).

**Table 3.** Growth performance of broiler ducks

Parameter		Group			SEM	P-value
		CON	CSMIX	CD+SG		
Viability (%)		98.33	98.33	96.67	1.008	0.761
BW (g)	Day 1	58.28	57.10	55.70	1.050	0.631
	Day 28	1871.92^a^	1850.62^a^	1467.37^b^	48.932	<0.001
	Day 49	3500.44^ab^	3574.87^a^	3367.78^b^	36.010	0.048
GR (%)	Days 1–28	187.92^a^	188.02^a^	185.38^b^	0.365	<0.001
	Days 29–49	60.73^b^	63.52^b^	78.59^a^	2.051	<0.001
	Days 1–49	193.45	193.70	193.49	0.108	0.625
BWG (g)	Days 1–28	1813.64^a^	1793.52^a^	1411.67^b^	48.539	<0.001
	Days 29–49	1628.51^b^	1724.25^b^	1900.41^a^	35.603	0.001
	Days 1–49	3442.15^ab^	3517.77^a^	3312.08^b^	35.587	0.047
FI (g)	Days 1–28	3444.53^b^	3957.65^a^	3353.18c	65.058	<0.001
	Feed	-	-	2429.18	-	-
	Silage	-	-	924.00	-	-
	Days 29–49	6395.16	6569.08	6346.75	51.793	0.410
	Feed	-	-	4890.70	-	-
	Silage	-	-	1456.05	-	-
	Days 1–49	9839.69^b^	10526.73^a^	9699.93^b^	136.838	0.023
	Feed	-	-	7319.88		
	Silage	-	-	2380.05		
FCR (kg/kg)	Days 1–28	1.90c	2.21^b^	2.38^a^	0.051	<0.001
	Days 29–49	3.93^a^	3.81^a^	3.34^b^	0.120	<0.001
	Days 1–49	2.86^b^	2.99^a^	2.93^a^	0.033	0.048
EBI (points)		241.82^ab^	265.41^a^	223.17^b^	5.820	0.004

1 Results are presented as mean value (n = 6 pens per group);

a,b… – when mean values have different letters in the same row, statistically significant differences were found (P-value < 0.05); CON – control group; CSMIX – group fed with a commercial diet with corn grain silage (ratio 80:20); CD+SG – group fed with restricted commercial diet and corn grain silage *ad libitum;* BW – body weight; GR – growth rate; BWG – body weight gain; FI – feed intake; FCR – feed conversion ratio; EBI – European broiler index

### Viscosity of intestinal digesta

Ducks in the CD+SG group had significantly higher jejunal viscosity than ducks in the CSMIX group (P-value = 0.005). It was noted that ducks in the CON and CSMIX groups had significantly lower ileal viscosity than their counterparts in the CD+SG group (P-value = 0.003) (Table 4).

**Table 4.** Broiler duck intestinal digesta viscosity

Digestive organ	Group			SEM	P-value
	CON (cP)	CSMIX (cP)	CD+SG (cP)		
Jejunum	3.68^ab^	3.25^b^	4.21^a^	0.128	0.005
Ileum	4.58^b^	4.82^b^	6.33^a^	0.220	0.003

a,b… – when mean values have different letters in the same row, statistically significant differences were found (P < 0.05); CON – control group; CSMIX – group fed with commercial diet with corn grain silage (ratio 80:20); CD+SG – group fed with restricted commercial diet and corn grain silage *ad libitum*

### Carcass tissue composition and meat quality

Significantly higher slaughter yield with offal was found in the CSMIX group compared to the CON group, while the CD+SG group did not differ significantly from either group (P-value = 0.036). The highest liver weight occurred in the CD+SG group, and this surplus was significant (P-value = 0.005). Significantly higher pectoral muscle weight (P-value = 0.031) and total muscle weight (P-value = 0.029) were found in the CON group than in the CD+SG group. Skin weight with subcutaneous fat (P-value = 0.030) and total fatness (P-value = 0.046) were significantly higher in the CD+SG group compared to the CON group. In contrast, the CSMIX group did not differ significantly from the other groups (Table 5).

**Table 5.** Broiler duck carcass composition

Parameter	Group			SEM	P-value
	CON	CSMIX	CD+SG		
Slaughter yield g/100 g pre-slaughter body weight	72.07	73.39	71.99	0.277	0.065
Slaughter yield with offal g/100 g pre-slaughter body weight	77.29^b^	78.81^a^	77.53^ab^	0.264	0.036
Heart g/100 g carcass with offal	0.69	0.68	0.69	0.013	0.934
Liver g/100 g carcass with offal	2.69^b^	2.67^b^	3.24^a^	0.084	0.005
Gizzard g/100 g carcass with offal	3.39	3.54	3.21	0.074	0.214
Neck g/100 g carcass with offal	7.15	7.07	7.01	0.147	0.932
Pectoral muscle g/100 g carcass	22.65^a^	21.71^ab^	20.94^b^	0.272	0.031
Leg muscle g/100 g carcass	13.44	13.21	13.14	0.199	0.824
Total muscle g/100 g carcass	36.09^a^	34.92^ab^	34.09^b^	0.317	0.029
Skin with subcutaneous fat g/100 g carcass	16.91^b^	18.92^ab^	19.66^a^	0.448	0.030
Abdominal fat g/100 g carcass	0.78	0.85	0.89	0.063	0.759
Total fatness g/100 g carcass	17.69^b^	19.77^ab^	20.55^a^	0.494	0.046
Wings with skin g/100 g carcass	12.27	11.88	11.83	0.138	0.366
Carcass remains g/100 g carcass	24.64	24.49	23.62	0.366	0.486

1 Results are presented as mean value (n = 12 ducks per group);

a,b… – when mean values have different letters in the same row, statistically significant differences were found (P-value < 0.05); CON – control group; CSMIX – group fed with a commercial diet with corn grain silage (ratio 80:20); CD+SG – group fed with restricted commercial diet and corn grain silage *ad libitum*

### Physicochemical properties of the pectoral and leg muscles

A significantly higher protein content was measured in the pectoral muscles of control group ducks than in these muscles of experimental group ducks (P-value = 0.001). In the pectoral muscles of the CON and CD+SG groups, significantly higher salt content (P-value < 0.001) was found. In CD+SG group birds’ pectoral muscles, significantly greater IMF content (P-value < 0.001) was present, and in the CSMIX group birds’ pectoral muscles significantly higher water content (P-value < 0.001) was observed. The leg muscles of ducks from the experimental groups were characterised by a significantly lower protein content (P-value = 0.033). More collagen was contained by leg muscles in the CON group than by these muscles in the CSMIX group, and this surplus was significant (P-value = 0.003) (Table 6).

**Table 6.** Physicochemical properties of broiler duck pectoral and leg muscles

	Parameter	Group			SEM	P-value
		CON	CSMIX	CD+SG		
Pectoral muscle	pH_24 hours_	5.98	6.01	5.60	0.320	0.428
	Drip loss %	2.09	2.20	1.82	0.250	0.623
	WHC % Colour	36.38	35.90	35.33	0.173	0.799
	^L*^	37.03	38.34	37.01	0.085	0.150
	^a*^	16.03	15.77	16.67	0.013	0.323
	^b*^	2.11	2.49	1.92	0.014	0.401
	Protein g/100 g muscle	23.01^a^	22.55^b^	22.26^b^	0.043	0.001
	Collagen g/100 g muscle	1.33	1.28	1.29	0.078	0.289
	Salt g/100 g muscle	0.38^a^	0.27^b^	0.37^a^	0.605	<0.001
	IMF g/100 g muscle	1.15^b^	1.07^b^	1.50^a^	0.454	<0.001
	Water g/100 g muscle	74.89^b^	75.46^a^	74.80^b^	0.324	<0.001
Leg muscle	WHC % Colour	32.04	31.27	28.73	0.232	0.061
	^L*^	36.73	37.38	38.32	0.143	0.368
	^a*^	15.46	15.00	14.95	0.052	0.788
	^b*^	2.83	2.93	3.09	0.033	0.903
	Protein g/100 g muscle	20.16^a^	19.40^b^	19.38^b^	0.138	0.033
	Collagen g/100 g muscle	1.95^a^	1.71^ab^	1.54^b^	0.131	0.003
	Salt g/100 g muscle	0.82	0.69	0.65	0.141	0.085
	IMF g/100 g muscle	5.16	5.52	4.98	0.161	0.266
	Water g/100 g muscle	72.51	72.41	72.71	0.621	0.650

1 Results are presented as mean value (n = 12 ducks per group);

a,b… – when mean values have different letters in the row, statistically significant differences were found (P-value < 0.05); CON – control group; CSMIX – group fed with a commercial diet with corn grain silage (ratio 80:20); CD+SG – group fed with restricted commercial diet and corn grain silage *ad libitum*; WHC – water holding capacity;

L* – lightness;

a* – redness;

b* – yellowness; IMF – intramuscular fat

### Fatty acids and vitamin content in pectoral muscles

In the pectoral muscles of ducks from the CD+SG group, statistically significantly different and the highest contents of C10:0 (P-value = 0.002), C12:0 (P-value < 0.001), C14:0 (P-value = 0.008), C14:1 (P-value = 0.032) and C15:0 (P-value = 0.015) were demonstrated. Statistically significantly lower contents of C:18:1n-9t were found in CON and CSMIX than in CD+SG (P-value = 0.003). Compared to the other groups, the control group had higher contents of C18:2n9t (P-value = 0.003) and C18:2n6c (P-value = 0.001). In the pectoral muscles of CON and CSMIX ducks, there were significantly higher contents of C20:2 (P-value < 0.001), C20:3n3 (P-value = 0.001) and C22:2 (P-value < 0.001). This tissue from the CD+SG group contained significantly more monounsaturated fatty acids (MUFA) (P-value = 0.007) and trans fatty acids (TFA) (P-value = 0.003). In the CON and CSMIX groups, a higher content of polyunsaturated fatty acids (PUFA) was noted (P-value < 0.001). Duck muscles from the CON and CSMIX groups were characterised by a significantly higher content of α-tocopherol (P-value = 0.039) and those from CON by content of β+γ tocopherol (P-value = 0.002) compared to the other groups (Table 7).

**Table 7.** Fatty acid profile and vitamin content of intramuscular fat in broiler duck pectoral muscles

Fatty acid	Group			SEM	P-value
	CON	CSMIX	CD+SG		
C10:0 (%)	0.10^ab^	0.09^b^	0.12^a^	0.004	0.002
C12:0 (%)	0.30^a^	0.21^b^	0.35^a^	0.014	<0.001
C14:0 (%)	1.33^b^	1.23^b^	1.70^a^	0.068	0.008
C14:1n-5 (%)(%)	0.19^ab^	0.17^b^	0.23^a^	0.009	0.032
C15:0 (%)	0.24^ab^	0.22^b^	0.29^a^	0.011	0.015
C16:0 (%)	21.94	22.02	22.64	0.170	0.185
C16:1n-7 (%)	1.23	1.19	1.41	0.044	0.089
C17:0 (%)	0.31	0.29	0.30	0.007	0.506
C17:1n-7 (%)	0.13	0.11	0.12	0.005	0.431
C18:0 (%)	14.47	14.30	13.99	0.135	0.339
C18:1n-9t (%)	19.22^b^	19.59^b^	21.93^a^	0.372	0.003
C18:1n-9c (%)	2.13	2.07	2.00	0.027	0.177
C18:2n-6t (%)	0.15^a^	0.12^b^	0.13^b^	0.004	0.003
C18:2n-6c (%)	18.77^a^	18.51^a^	16.85^b^	0.242	0.001
C18:3n-3 (%)	0.54	0.51	0.53	0.010	0.564
C20:0 (%)	0.25^a^	0.24^ab^	0.22^b^	0.005	0.041
C20:1n-9 (%)	0.48	0.46	0.45	0.011	0.666
C20:2 (%)	1.25^a^	1.24^a^	0.95^b^	0.031	<0.001
C20:3n-3 (%)	1.17^a^	1.20^a^	1.02^b^	0.022	0.001
C20:4n-6 (%)	11.66	12.06	11.05	0.243	0.234
C20:5n-3 (%)	0.09	0.10	0.10	0.003	0.456
C21:0 (%)	0.12^a^	0.08^b^	0.13^a^	0.006	<0.001
C22:2 (%)	0.70^a^	0.75^a^	0.50^b^	0.022	<0.001
C22:6n-3 (%)	0.62	0.61	0.62	0.017	0.986
C24:0 (%)	0.06	0.06	0.07	0.001	0.208
C24:1n-9 (%)	2.56	2.57	2.32	0.049	0.057
SFA (%)	39.12	38.73	39.80	0.198	0.078
MUFA (%)	25.94^b^	26.16^b^	28.46^a^	0.378	0.007
PUFA (%)	34.94^a^	35.11^a^	31.74^b^	0.411	<0.001
TFA (%)	19.37^b^	19.71^b^	22.05^a^	0.371	0.003
α-tocopherol μg/100g	319.09^a^	317.57^a^	274.47^b^	8.265	0.039
β+γ tocopherol μg/100g	41.33^a^	34.91^b^	32.68^b^	1.086	0.002
Retinol μg/100g	10.28	9.26	10.72	0.308	0.140

a,b… – when mean values have different letters in the row, statistically significant differences were found (P-value < 0.05); CON – control group; CSMIX – group fed with commercial diet with corn grain silage (ratio 80:20); CD+SG – group fed with restricted commercial diet and corn grain silage *ad libitum*; C10:0 – decanoic acid; C12:0 – dodecanoic acid, C14:0 – tetradecanoic acid; C14:1n-5 – cis-9-tetradecenoic acid; C15:0 – pentadecanoic acid; C16:0 – hexadecanoic acid; C16:1n-7 – cis-9-hexadecenoic acid; C17:0 – heptadecanoic acid; C17:1n-7 – cis-10-heptadecenoic acid; C18:0 – octadecanoic acid; C18:1n-9t – trans-9-octadecenoic acid; C18:1n-9c – cis-9-octadecanoic acid; C18:2n-6t – trans-9,12-octadecadienoate acid; C18:2n-6c – cis,cis-9,12-octadecadienoic acid; C18:3n-3 – cis,cis,cis-9,12,15-octadecatrienoic acid; C20:0 – arachidic acid; C20:1n-9 – cis-11-eicosenoic acid; C20:2 – cis-11,14-eicosadienoic acid; C20:3n-3 – cis-11,14,17-eicosatrienoic acid; C20:4n-6 – arachidonic acid; C20:5n-3 – eicosapentaenoic acid; C21:0 – heneicosanoic acid; C22:2 – docosadienoic acid; C22:6n-3 – cis-4,7,10,13,16,19-docosahexaenoic acid; C24:0 – lignoceric acid; C24:1n-9c – nervonic acid; SFA – saturated fatty acids (C10:0, C12:0, C14:0, C15:0, C16:0, C17:0, C18:0, C20:0, C21:0, C22:0, C23:0 and C24:0); MUFA – monounsaturated fatty acids (C14:1n-5, C16:1n-7, C17:1n-7, C18:1n-9t, C18:1n-9, C20:1n-9 and C24:1n-9); PUFA – polyunsaturated fatty acids (C18:2n-6t, C18:2n-6c, C18:3n-3, C20:2, C20:3n-3, C20:4n-6, C20:5n-3, C22:2, C22:6n-3 and C24:6n-3); TFA – trans fatty acids (C18:1n-9t and C18:2n-6t)

### Fatty acids and vitamin content in leg muscles

In the leg muscles of ducks from the CD+SG group, significantly higher contents of C10:0 (P-value < 0.001), C12:0 (P-value < 0.001), C14:0 (P-value < 0.001), C14:1 (P-value < 0.001), C15:0 (P-value < 0.001), C16:0 (P-value < 0.001) and C17:1 (P-value = 0.005) were found compared to these fatty acid contents in the leg muscles of ducks from the CON and CSMIX groups. Similar results were obtained for C20:5n-3 (P-value < 0.001), C22:2 (P-value < 0.001) and C24:0 (P-value =0.001). The lowest content of C18:2n-6t was detected in the CSMIX group (P-value = 0.005), and the lowest contents of C18:2n-6c (P-value < 0.001), C18:3n-3 (P-value < 0.001) and C20:2 (P-value = 0.008) in the CD+SG group. This tissue from the CON and CSMIX groups was characterised by a significantly lower saturated fatty acid (SFA) content than the same tissue from the CD+SG groups (P-value < 0.001). The opposite situation occurred in the case of PUFA (P-value < 0.001). In the leg muscles of the control group, there was a significantly higher content of β+γ tocopherol than in those of the 80:20-proportioned feed group (P-value = 0.049) (Table 8).

**Table 8.** Fatty acid profile and vitamin content of broiler duck leg muscles

Fatty acid	Group			SEM	P-value
	CON	CSMIX	CD+SG		
C10:0 (%)	0.10^b^	0.09^b^	0.16^a^	0.008	<0.001
C12:0 (%)	0.31^b^	0.28^b^	0.48^a^	0.018	<0.001
C14:0 (%)	1.31^b^	1.26^b^	2.14^a^	0.077	<0.001
C14:1 (%)	0.20^b^	0.20^b^	0.31^a^	0.011	<0.001
C15:0 (%)	0.24^b^	0.21^b^	0.35^a^	0.012	<0.001
C16:0 (%)	19.49^b^	19.28^b^	21.13^a^	0.209	<0.001
C16:1n-7 (%)	1.87	1.99	2.03	0.038	0.237
C17:0 (%)	0.28	0.26	0.31	0.008	0.073
C17:1n-7 (%)	0.13^b^	0.13^b^	0.15^a^	0.004	0.005
C18:0 (%)	13.87	13.48	13.86	0.179	0.607
C18:1n-9t (%)	25.69	27.49	26.95	0.363	0.112
C18:1n-9c (%)	1.97	1.90	17.15	5.057	0.377
C18:2n-6t (%)	0.18^a^	0.15^b^	0.18^a^	0.004	0.005
C18:2n-6c (%)	19.29^a^	17.89^a^	14.54^b^	0.451	<0.001
C18:3n3 (%)	0.80^a^	0.69^b^	0.62^b^	0.017	<0.001
C20:0 (%)	0.17	0.15	0.18	0.005	0.084
C20:1n-9 (%)	0.46	0.46	0.45	0.008	0.777
C20:2 (%)	0.83^a^	0.76^ab^	0.67^b^	0.021	0.008
C20:3n-3 (%)	0.67	0.67	0.64	0.011	0.362
C20:4n-6 (%)	8.52	9.02	8.74	0.168	0.489
C20:5n-3 (%)	0.09^b^	0.10^b^	0.15^a^	0.006	<0.001
C21:0 (%)	0.12	0.13	0.15	0.006	0.077
C22:2 (%)	0.34^b^	0.32^b^	0.45^a^	0.013	<0.001
C22:6n-3 (%)	0.66^ab^	0.64^b^	0.77^a^	0.021	0.024
C24:0 (%)	0.05^b^	0.04^b^	0.06^a^	0.003	0.001
C24:1n-9 (%)	2.36	2.42	2.41	0.045	0.842
SFA (%)	35.95^b^	35.18^b^	38.81^a^	0.354	<0.001
MUFA (%)	32.69	34.59	34.27	0.366	0.071
PUFA (%)	31.37^a^	30.24^a^	26.74^b^	0.495	<0.001
TFA (%)	25.88	27.65	27.13	0.360	0.117
α-tocopherol μg/100g	229.91	220.82	194.28	6.558	0.066
β+γ tocopherol μg/100g	32.94^a^	25.48^b^	28.64^ab^	1.310	0.049
Retinol μg/100g	8.11	8.61	9.10	0.189	0.100

a,b… – when mean values have different letters in the row, statistically significant differences were found (P-value < 0.05); CON – control group; CSMIX – group fed with commercial diet with corn grain silage (ratio 80:20); CD+SG – group fed with restricted commercial diet and corn grain silage *ad libitum;* SEM – standard error of the mean; C10:0 – decanoic acid; C12:0 – dodecanoic acid, C14:0 – tetradecanoic acid; C14:1n-5 – cis-9-tetradecenoic acid; C15:0 – pentadecanoic acid; C16:0, hexadecanoic acid; C16:1n-7, cis-9-hexadecenoic acid; C17:0, heptadecanoic acid; C17:1n-7 – cis-10-heptadecenoic acid; C18:0 – octadecanoic acid; C18:1n-9t – trans-9-octadecenoic acid; C18:1n-9c – cis-9-octadecanoic acid; C18:2n-6t – trans-9,12-octadecadienoate acid; C18:2n-6c – cis,cis-9,12-octadecadienoic acid; C18:3n-3 – cis,cis,cis-9,12,15-octadecatrienoic acid; C20:0 – arachidic acid; C20:1n9 – cis-11-eicosenoic acid; C20:2 – cis-11,14-eicosadienoic acid;; C20:3n-3 – cis-11,14,17-eicosatrienoic acid; C20:4n-6 – arachidonic acid; C20:5n3 – eicosapentaenoic acid; C21:0 – heneicosanoic acid; C22:2 – docosadienoic acid; C22:6n-3 – cis-4,7,10,13,16,19-docosahexaenoic acid; C24:0 – lignoceric acid; C24:1n-9c – nervonic acid; SFA – saturated fatty acids (C10:0, C12:0, C14:0, C15:0, C16:0, C17:0, C18:0, C20:0, C21:0, C22:0, C23:0 and C24:0); MUFA – monounsaturated fatty acids (C14:1n-5, C16:1n-7, C17:1n-7, C18:1n-9t, C18:1n-9, C20:1n9 and C24:1n-9); PUFA – polyunsaturated fatty acids (C18:2n-6t, C18:2n-6c, C18:3n-3, C20:2, C20:3n-3, C20:4n-6, C20:5n-3, C22:2, C22:6n-3 and C24:6n-3); TFA – trans fatty acids (C18:1n-9t and C18:2n-6t)

### Estimated costs of feeding

The costs of feeding ducks in the first rearing period in the CD+SG group were significantly lower than in the other groups (P-value < 0.001). In the second feeding period and throughout the whole rearing period, feeding costs were significantly lower in both experimental groups compared to the control group (P-value < 0.001). (Table 9).

**Table 9.** Estimated costs of feeding ducks

Item per 1 duck (PLN)	Group			SEM	P-value
	CON	CSMIX	CD+SG		
Starter costs (days 1–28)	8.54^a^	8.62^a^	6.92^b^	0.191	<0.001
Grower costs (days 29–49)	15.16^a^	13.73^b^	13.00^c^	0.229	<0.001
Total cost of feeding	23.70^a^	22.35^b^	19.92^c^	0.386	<0.001

a,b… – when mean values have different letters in the row, statistically significant differences were found (P-value < 0.05); CON – control group; CSMIX – group fed with commercial diet with corn grain silage (ratio 80:20); CD+SG – group fed with restricted commercial diet and corn grain silage *ad libitum*

## Discussion

The highest BW (day 49) and BWG (days 1–49) were determined in the CSMIX group; however, they were not statistically different from the parameters in the CON group. Ducks from the CSMIX group were characterised by the highest FI, which impacted the final FCR at 2.99 kg/kg. Ridla *et al*. (48) used silage in the diet of male Alabio ducks, which was prepared from cassava, cassava leaves, and soybean. They observed a higher BWG in the experimental group where silage with 50% water content was used. In contrast, Sulaiman *et al*. (51) showed no significant changes in the production results of Pekin ducks fed sago pith silage at 5, 10, 15 and 20% from the 3^rd^ to the 8^th^ week of rearing. In another contrast to our study, Zaremba *et al*. (64) also reported that an alternative feeding strategy for broiler ducks based on limited feeding with commercial diet and residual nutritional need met by beet pulp silage or maize silage did not affect the final BW or BWG. The same researchers fed ducks beet pulp silage (mixed with a commercial diet in a ratio of 30:70) and noted consumption of significantly more feed by these ducks than by ducks provided a commercial diet in a limited proportion and beet pulp silage *ad libitum* (63).

The silage production process, particularly the silage additives used, determines its usefulness and biological activity (16). Organic acids such as formic, propionic or acetic acid maintain the stability of the silage process and create a suitable environment for developing desirable microorganisms (16, 23). They are beneficial because they acidify the diet content and improve digestion of nutrients, affecting BW, FCR and FI (49). Elnagar and El-Maaty (12) confirmed that organic acids (formic and citric acids) are suitable additives for the broiler duck diet, attested to by better body weight gain and support of the anatine immune system. In addition, the effectiveness of formic, propionic, and lactic acids was noted in increasing the height of intestinal villi and reducing the occurrence of pathogenic bacteria such as *Escherichia coli* and *Clostridium perfringens* in Khaki Campbell ducks (26). According to Carvalho-Estrada *et al*. (8), the bacterial community of maize silage is dominated by *Lactobacillus* and *Acetobacter* bacteria. The former are frequent constituents of probiotic preparations in poultry nutrition (45, 47). Therefore, maize silage has probiotic properties. Various mechanisms of action of probiotic microorganisms, including adhesion to the intestinal epithelium, competition for the habitat, and production of bacteriocins and lactic acid, were stated to promote better BWG and robust health (1). The favourable BW and BWG results for the CSMIX group may be confirmation of these mechanisms in action.

It is suggested that the higher body weight of birds in the CSMIX group than that of birds in the CD+SG group elevated the EBI (despite the similar final FCR). In the study by Zaremba *et al*. (63), the highest and a significantly different EBI was calcualted for ducks fed with beet pulp silage *ad libitum* compared to the group where a mix of a commercial diet and silage (70: 30) was used and to the control group. Biesek *et al*. (6) conducted research on a possible effect on production efficiency of alternative feeding of broiler ducks by replacing the commercial diet with wheat grain in 10, 20 and 40% proportions in the last week of rearing. The authors found no significant differences in the EBI between the groups. However, it is worth noting that the EBI in both cited studies did not exceed 210, whereas in our study, the CSMIX group attained 261.01. It indicates a favourable average daily gain to FCR ratio.

The highest viscosity of jejunal and ileal contents was shown in the group where ducks had unlimited access to silage (4.21 cP and 6.33 cP, respectively). Fibre content in the feed is one of the main factors that affect viscosity, in the findings of Tejeda and Kim (53). The authors showed that 8% fibre in the diet significantly reduced BWG and villus height and crypt depth in the small intestine, and also increased the viscosity of intestinal contents compared to groups where 4% fibre was used. Lee *et al*. (28) also showed a negative effect of higher intestinal viscosity on BWG. This is due to the feed’s lower digestibility and the consuming organism’s inefficient use of nutrients (58). Higher viscosity slows the rate of digesta flow. The effects are lower oxygen levels and the formation of acetic acid (32). Increased intestinal viscosity may confirm the presence of soluble fibre fractions in the silage (53). Ensiled corn grain contains a large amount of crude fibre, ADF, NDF and ADL, which would explain the significant differences in viscosity and BW, especially in the CD+SG group.

A significantly higher slaughter yield with offal was achieved in the CSMIX group compared to the CON group. Ducks in the control group had significantly higher pectoral and total muscle weight than ducks in the group which consumed ensiled maize grain *ad libitum*. Significantly lower total fatness was demonstrated in the CON group than in the CD+SG group. Aslan and Ozturk (3) analysed the possibility of feeding indigenous male Turkish geese with maize silage mixed with feed at different levels (10:90, 20:80, 30:70 and 40:60). They did not find differences in slaughter yield, gizzard or liver weight. Similar results were presented by Wang *et al*. (57) in terms of carcass yield, pectoral muscle yield, leg muscle yield, liver yield and heart yield in Hortobágy geese fed whole-plant silage. However, Kokoszyński *et al*. (25) showed that carcasses of White Kołuda W31 geese fed restricted amounts of a commercial diet and maize silage *ad libitum* were characterised by significantly higher dressing percentages compared to the control group (74.7% *vs* 65.0%).

Bile is produced in the liver hepatocytes and then stored in the gallbladder. It contains essential components for body fat digestion, such as bile salts and phospholipids (46). Ducks in the CD+SG group potentially ate the most ensiled grain, which contained almost twice as much crude fat as the commercial starter and grower feed. To enhance fat breakdown and increase bile production, liver cells can expand. Considering the likely greatest fat consumption by CD+SG ducks, the most extensive liver cell expansion was in these ducks, consequently increasing their liver weight. The optimal dietary metabolisable-energy-to-crude-protein ratio is crucial in obtaining high weight gain and good meat quality. Low metabolisable energy in the diet limits abdominal fat accumulation, which high energy content does not (9). The CD+SG group’s lower pectoral muscle and total muscle weight, as well as higher skin with subcutaneous fat and total fatness, may be due to the low crude protein content of the silage and its higher fat content compared to the commercial diet.

The pectoral and leg muscles of the ducks in the experimental groups had a significantly lower protein content than those of the ducks in the control group. The pectoral muscles of the ducks in the CD+SG group contained significantly more IMF. These muscles of the ducks from the CSMIX group had significantly higher salt and water content. The diet’s quality and quantity of crude protein affect protein metabolism and muscle content (55). Marcu *et al*. (30) reported that a feed protein and energy content higher than the standard by 10% enhanced the protein and fat content in chicken pectoral muscles compared to these muscles of chickens receiving feed with these contents lower than the standard by 10%. According to Infante-Rodríguez *et al*. (20), of four broiler chicken diets with the same crude protein content of 21.4% in the starter formulation and 18.7% in the finisher formulation, the diet with the highest content of apparent metabolisable energy (3,080 for starters and 3,160 kcal/kg for finishers) gave the lowest content of ether extract in the pectoral muscles. Moreover, no changes in the chemical composition of leg muscles were observed. Puchajda *et al*. (44) showed significantly higher protein contents in the pectoral muscles of geese fed with grass and clover silage than in the muscles of geese fed steamed potato silage. Wang *et al*. (56) observed that paper mulberry silage in the diet of Yangzhou geese improved the sensory and nutritional quality of meat as a result of good protein availability.

Ensiled corn grain was characterised by a higher proportion of PUFA and a lower proportion of SFA than commercial feed (quantitatively). Significantly lower contents of α-tocopherol and retinol were also found. Lower SFA content was evident in the leg muscles of ducks from the CON and CSMIX groups than in these muscles of CD+SG-group ducks. The fatty acid profile in meat can be modified by nutrition (50). Kowalska *et al*. (27) showed a significantly higher content of n-3 fatty acids (P-value = 0.006) in the subcutaneous fat of Pekin ducks after providing feed containing 60.10% yellow lupin and 14.00% rapeseed meal as an alternative protein source to post-extraction soybean meal. In the study by Huo *et al*. (19), keeping ducks in an integrated rice-duck farming system positively affected the fatty acid composition of meat compared to the floor pen–rearing system. The SFA content (C12:0, C14:0, C16:0, C18:0 and C21:0), MUFA content (C16:1, C17:1, and C18:1) and PUFA content (C22:2, C18:2n-6 and C22:6n-3) were higher. A high SFA content in the diet negatively impacts the circulatory system (29) and increases the risk of other diseases (22). Therefore, the presented results in the CSMIX group suggest that consumption of the meat of ducks so fed would be beneficial to health. Fats rich in unsaturated fatty acids are better digested by birds than fats high in saturated fatty acids (2). Therefore, the more suitable PUFA to SFA ratio in the experimental diet (CSMIX) could have contributed to the higher proportion of PUFA in the pectoral muscles of ducks from this group. The oxidation products of unsaturated fatty acids are hexanaldehydes and nonanaldehydes, which define the aroma of meat to the greatest extent, as stated by Wasilewski *et al*. (59) Their content may form attractive sensory characteristics in duck meat when it is high.

Feed costs can constitute up to 70% of total poultry production costs, which indicates feed’s high importance as a delimiter of production profitability and the wisdom of accounting for it in estimating potential revenues (54). Unconventional methods of poultry feeding based on cheaper substitutes for commercial diets can set more favourable economic parameters for production. The costs of feeding ducks with ensiled maize grain (the CD+SG group) were significantly lower, and lighter budgetary burdens were borne feeding both experimental groups with grower feed. Wu *et al*. (61) found a favourable net present value in an alternative system of duck rearing in corn fields compared to traditional indoor production. Reduction of feed costs is possible by keeping ducks in rice fields and using the restrictive feeding method used in this study (4). Biesek *et al*. (6) also reduced feed costs per duck during 49 days of rearing using partial replacement of feed mixture with wheat at 20% and 40% by PLN 1.98 and 2.25, respectively, compared to costs of feed as commercial granulated products.

## Conclusion

Ducks from the CSMIX group had comparable BWG to those in the CON group, but significantly higher FI and FCR. Increased dietary fibre content in ensiled maize grain determined the viscosity of intestinal content, which translated into production results. Characteristic features of the chemical composition of ensiled maize grain mainly increased energy and fat and decreased protein in the feed. These changes contributed to differences in carcass composition and physicochemical quality features of meat. A similar carcass composition to ducks from the control group characterised ducks provided feed mixed with ensiled maize grain. Reduction in the commercial feed proportion and giving silage *ad libitum* negatively affected the nutritional value of meat, in particular the content of protein and fat. Pectoral and leg muscles of ducks in the exclusively granulated-feed–sustained and 80:20 groups revealed a more beneficial fatty acid profile than those of birds fed restrictively, considering PUFA and SFA. In the experimental groups, total feeding costs were lower. An alternative feeding strategy for broiler ducks based on a commercial diet and ensiled maize grain (mixed 80:20) may be recommended to achieve higher body weight gains and obtain meat with a beneficial fatty acid profile while reducing feed costs.
